# Modeling neuron growth using isogeometric collocation based phase field method

**DOI:** 10.1038/s41598-022-12073-z

**Published:** 2022-05-17

**Authors:** Kuanren Qian, Aishwarya Pawar, Ashlee Liao, Cosmin Anitescu, Victoria Webster-Wood, Adam W. Feinberg, Timon Rabczuk, Yongjie Jessica Zhang

**Affiliations:** 1 Department of Mechanical Engineering, Carnegie Mellon University, Pittsburgh, 15213 USA; 2 School of Mechanical Engineering, Purdue University, West Lafayette, 47907 USA; 3 Department of Biomedical Engineering, Carnegie Mellon University, Pittsburgh, 15213 USA; 4 Department of Materials Science and Engineering, Carnegie Mellon University, Pittsburgh, 15213 USA; 5 Institute of Structural Mechanics, Bauhaus-Universität Weimar, 99423 Weimar, Germany

**Keywords:** Computational models, Biophysical models

## Abstract

We present a new computational framework of neuron growth based on the phase field method and develop an open-source software package called “NeuronGrowth_IGAcollocation”. Neurons consist of a cell body, dendrites, and axons. Axons and dendrites are long processes extending from the cell body and enabling information transfer to and from other neurons. There is high variation in neuron morphology based on their location and function, thus increasing the complexity in mathematical modeling of neuron growth. In this paper, we propose a novel phase field model with isogeometric collocation to simulate different stages of neuron growth by considering the effect of tubulin. The stages modeled include lamellipodia formation, initial neurite outgrowth, axon differentiation, and dendrite formation considering the effect of intracellular transport of tubulin on neurite outgrowth. Through comparison with experimental observations, we can demonstrate qualitatively and quantitatively similar reproduction of neuron morphologies at different stages of growth and allow extension towards the formation of neurite networks.

## Introduction

**Figure 1:**
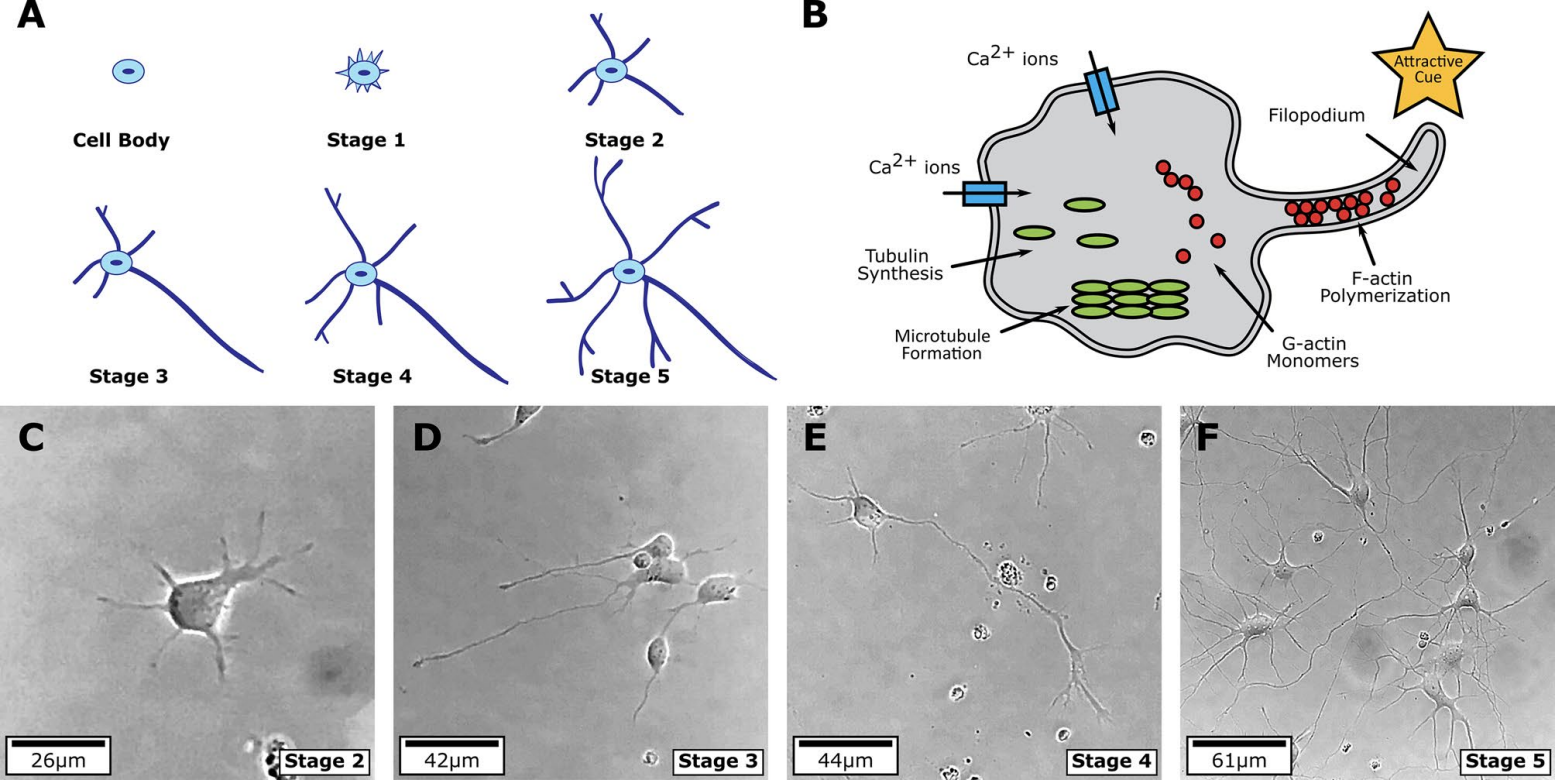
Five stages of neuron growth from initiation to maturation and adapted schematic diagram^15,16^ showing the neurite elongation (stages 2–5) in the presence of attractive cues. (**A**) The stages observed are lamellipodia formation (few hours), initial outgrowth of neurites (${\approx }$ 1 day), axon differentiation (${\approx }$ 1.5 days), dendrite formation (${\approx }$ 4 days) and neuron maturation (${\gtrsim }$ 7 days)^1^. (**B**) Tubulin is produced at the cell body and transported via active transport and diffusion to the neurite ends^10^. The assembly of tubulin at the neurite tips leads to neurite elongation. (**C**–**F**) Experimental images of neuron culture corresponding to growth stages 2 to 5.

Neuron growth is a complex phenomenon which consists of different stages of development (see Fig. 1A,C–F). In stage 1, there is lamellipodia formation from an initial spherical cell. The lamellipodia result into several neurites of approximately similar lengths in stage 2. Next, the longest neurite differentiates into an axon in stage 3 after which the remaining neurites start to grow leading to dendrite formation in stage 4. Finally, in stage 5 the neuron maturation occurs. The entire process takes several days^1–4^. Mathematical modeling of the early stages (stages 1–3) such as lamellipodia formation, initial neurite outgrowth and axon differentiation have been proposed^4–6^. Starting with a spherical cell, there is an influx of sodium and calcium ions through the cell membrane^2,5,7^. There is an increase in the influx of calcium ions due to bulges on the surface, leading to lamellipodia formation and initial neurite outgrowth. After the initiation process the further outgrowth of neurites is assumed to be carried out by the transport of a chemical produced in the cell body to the neurite ends^4^. The longest neurite has higher consumption of the chemical, resulting in its higher growth rate as compared to the other neurites. This stage is referred to as axon differentiation. During stages 4 and 5, there is further elongation and branching of neurites leading to dendrite formation^7^. The construction of cytoskeleton, which is an integral part of the cell, leads to neurite elongation^8,9^. A schematic representation of neurite elongation, which takes place during stages 2–5 through cytoskeleton construction, is shown in Fig. 1B. The extension of the cytoskeleton takes place through microtubule assembly at the neurite ends. Microtubules are transported from the cell body and assembled at the neurite ends. Thus, the assembly rate of microtubules is a function of the amount of tubulin present at the growing tip of the neurite^7,10,11^. Equations to model tubulin concentration along the length of single unbranched axon were proposed^10,12^. These equations consider the active transport and diffusion of tubulin from the cell body to the neurite tip. During the maturation stage (stage 5), the creation of the dendritic structures for different neuron types^11,13,14^ is considered.

In the computational modeling for different stages of neuron growth, most models focus on certain stages such as neurite initiation^5^, axon growth^17–20^, or growth cone locomotion^21^. These models help in gaining a better understanding of specific stages of neuron growth. However, they can only model one stage at a time. In addition, most of the composite models are limited to one-dimensional geometry^22^. Computational models using the phase field method have been applied to study certain stages of neuron growth^15,22,23^. For example, phase field methods have been extensively used to study moving boundary and sharp interface problems^24,25^. These methods have also been implemented to study different biological phenomena^26–28^. A phase field model for 2D axon extension was proposed^23^. This model described a good preliminary study for neuron growth through the application of a modified Kobayashi-Warren-Carter (KWC) model^29–33^. The computational model was divided into three stages: initial outgrowth of numerous neurites of almost equivalent lengths, decrease in the number of neurites extending and neurite retraction in a transition model, and single axon extension. While the model could capture the axon extension in the presence of nerve growth factor in the extracellular medium, it does not consider intracellular factors such as the active transport and diffusion of tubulin to model axon elongation and differentiation. Moreover, it also manually applies constraints to simulate axon differentiation. This process is not automatic as it fails to consider the intrinsic factors one of which being the rate-limited consumption of tubulin.

High-fidelity geometric modeling for complex neuron structures has been an ongoing challenge in the field of computational biology^34,35^. Various spline-based neuron image segmentation techniques have been proposed^36,37^. Conventional finite element analysis (FEA) lacks the ability to handle complex neuron geometry effectively without extensive discretizations. Isogeometric analysis (IGA) was introduced to bridge the gap between geometry and analysis^38^. IGA directly utilizes smooth high-order spline geometry to produce accurate analysis results while significantly lowering the number of degrees of freedom (DOF). In addition, isogeometric collocation method is shown to significantly speed up simulations^39^, and successfully combine geometrical flexibility, accuracy, and simplicity^40^.

In our proposed work, we define the phase field formulation of neuron morphogenesis based on dendritic solidification^23,41^ to model the lamellipodia formation and initial neurite outgrowth stages. We incorporate the effect of intrinsic factors such as the effect of tubulin concentration in order to model the axon differentiation stage automatically. The proposed model can also capture the dendrite formation stage. The main contributions of the proposed mathematical model include:

- Development of a novel phase field model that describes primarily stages 1 to 4 of neuron growth in the presence of intracellular tubulin concentration, including lamellipodia formation, initial neurite outgrowth, axon differentiation, and dendrite formation.
- Development of a neuron growth simulation model based on phase-field method using multi-resolution isogeometric collocation method.
- Comparison of different metrics such as segment length and turning angle of each segment with experimental images of rat hippocampal neurons is demonstrated to show a similar reproduction of end-stage neuron morphology.
- Extension of the phase field model to carry out the growth of neural circuits, where growth of neurons towards each other is observed, leading to neurite interaction and complex neural networks.

## Results

### Simulations of single and multiple neuron configurations

**Figure 2:**
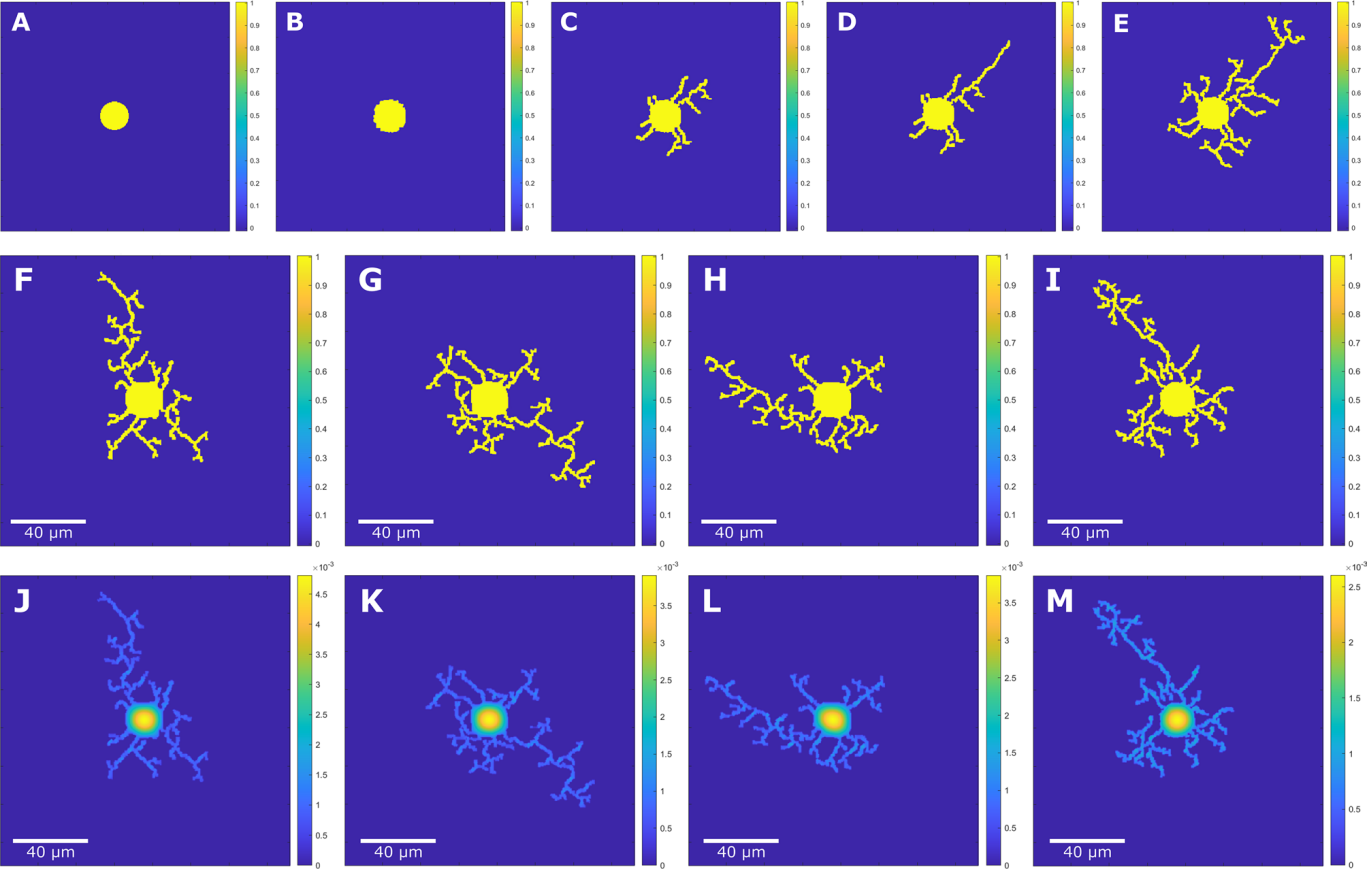
Multi-stage neuron growth phase field result and four single neuron growth results. The phase field variable field $\phi$, is shown in (**A**–**I**). (**A**) Initial circular cell at the center of the domain of grid size $380 \times 380$ (0 iteration). (**B**) Stage 1 lamellipodia formation (500 iterations) and (**C**) Stage 2 initial outgrowth of neurites (10,500 iterations) where the neurites grow to similar length with no constraints applied. (**D**) The longest neurite grows out further than the rest of the neurites using an extracellular cue-based energy activation zone approach, leading to stage 3 axon differentiation (28,500 iterations). (**E**) Stage 4 dendrite formation (35,000 iterations) is observed by allowing the neurite extension and branching for more iterations. (**F**–**I**) shows four phase field model results of single neuron growth using different $\theta$ initializations using grid size of $383 \times 383$, $303 \times 303$, $363 \times 363$, and $343 \times 343$ respectively. Each case exhibits growth behavior from stage 1 lamellipodia formation to stage 4 dendrite formation with observable stage 3 axon differentiation. The axon growth exhibits similar angle changes with experimental data-based extracellular cue placement. (**J**–**M**) Intracellular tubulin concentration field is shown for the corresponding phase field results.

**Figure 3:**
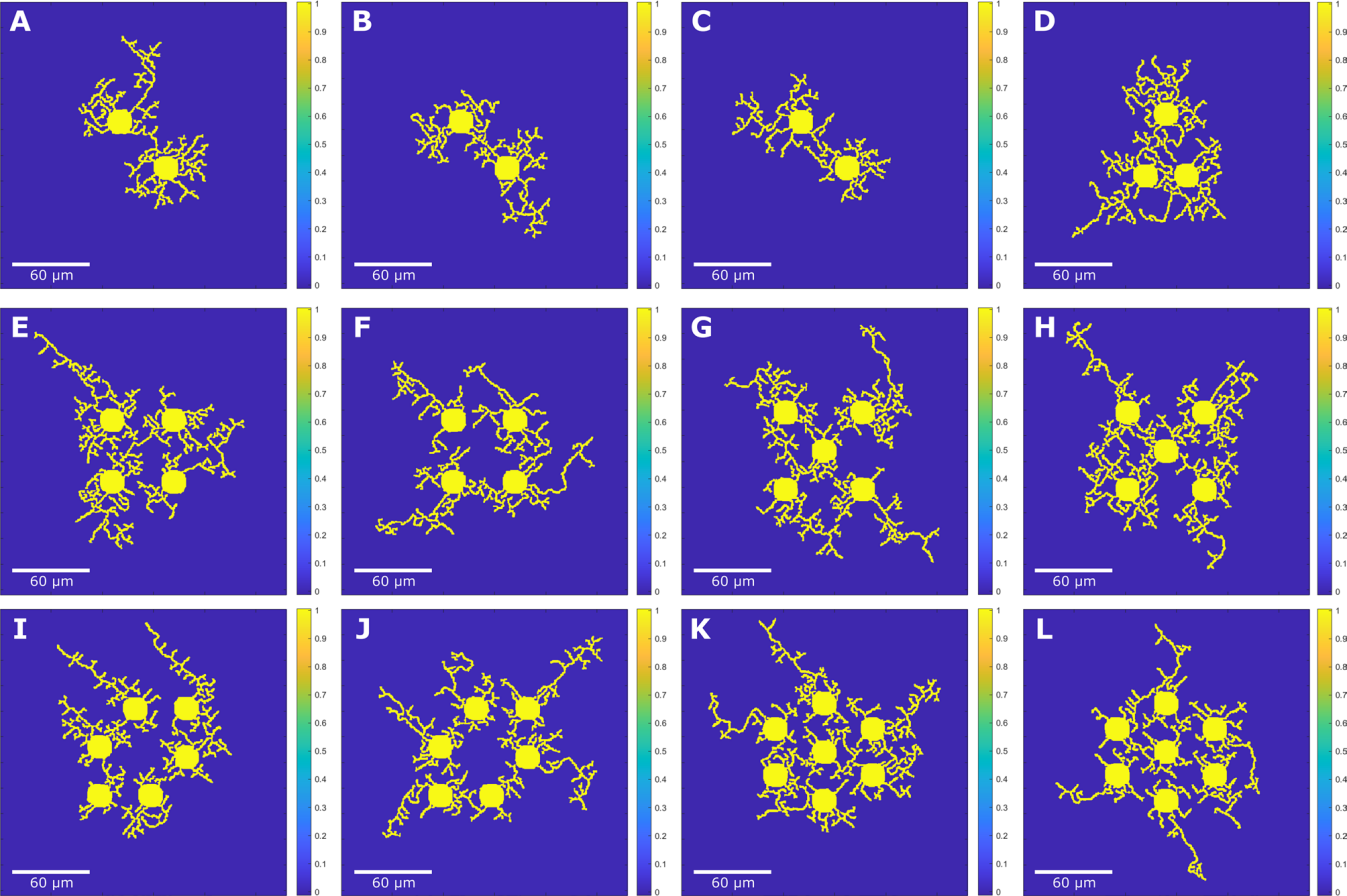
Phase field model results of multiple neurons with all 4 growth stages using different random $\theta$ initializations with the grid size of $560 \times 560$. Neuron growth simulation with neurite interaction is shown for (**A**–**C**) 2 neurons ($453 \times 453$, $393 \times 393$, $383 \times 383$), (**D**) 3 neurons ($353 \times 353$), (**E**,**F**) 4 neurons ($493 \times 493$, $463 \times 463$), (**G**,**H**) 5 neurons ($523 \times 523$, $533 \times 533$), (**I**,**J**) 6 neurons ($533 \times 533$, $493 \times 493$), and (**K**,**L**) 7 neurons ($553 \times 553$, $543 \times 543$). Using self-intersection check, multiple neurons exhibit neurite interactions with each other while preventing interactions between neurites belonging to the same neuron, demonstrating that the model can handle multi-neuron interactions during simulation. Simulations are plotted using the same domain size and scale bars are calculated based on cell body diameter observed in experimental images shown in Fig. 4.

We show the results of single neuron cell growth obtained from the presented neuron growth model in Fig. 2A–E. Through IGA collocation, we compute the gradient of $\phi$ accurately using $C^{2}$ continuous B-spline basis functions. Starting from a circular cell, multiple neurites emerge spontaneously from the cell surface. This corresponds to the lamellipodia formation stage (stage 1). Similar results can be seen for the non-constraint model^23^. Figure 2F–M shows four single neuron growth cases where stages 1–4 are captured along with corresponding tubulin concentration distribution. The diffusion of tubulin from the cell body to the neurite tips is shown. In the absence of competitive effects, all the neurites grow at uniform rates to similar lengths. The growth rate depends on the concentration of tubulin and the degree of anisotropy. Thus, we observe a direct correlation between neuron growth with intracellular factors. To achieve the axon differentiation stage (stage 3), the longest neurite is detected, and the *E* value in the energy activation zone for all the other neurites other than the longest one is set as 0. In Fig. 3, we demonstrate the flexibility of phase-field model to study neuron growth of multiple neuron networks with neurons arranged in multiple configurations. Each neuron exhibits differential growth of the axon, dendritic branching and interactions between neurites.

### Comparison of experimental culture image and neuron growth model results

In order to draw a comparison of the proposed phase field model results with experimental observations of growing neurons, primary embryonic day 18 (E18) rat hippocampal neurons were cultured and imaged over 20 days in vitro (DIV). Individual neurites from after 20 DIV were then analyzed to extract length and angle information for the comparison.

#### Neuron cell culture

Primary rat hippocampal neurons (RHiN) were cultured per manufacturer’s protocol^42^ and imaged regularly from 0-20 DIV. Briefly, cryopreserved primary RHiN (A36513, Gibco, USA) were thawed, plated, and maintained on a cell-culture-treated 48-well plate (150687, Nunc, USA). To promote cell adhesion the culture surfaces were coated with a poly-d-lysine (A3890401, Gibco, USA) solution diluted in 0.5 M borate buffer (PI28341, ThermoScientific, USA) to a concentration of 50 ${{\mathrm{g}}/\upmu {\mathrm{L}}}$, as per the manufacturer’s protocol^42^. Plates were incubated at room temperature for one hour during coating, after which the solution was removed and each well was rinsed with Dulbecco’s phosphate-buffered saline (14190144, Gibco, USA)^42^. The well plate was then allowed to dry under sterile conditions at room temperature for two hours. Prior to culture, plates were wrapped with Parafilm (Bemis, PM999, USA) and stored overnight at 6${^{\circ }}$^42^.

For culture, cryopreserved RHiNs were thawed rapidly at 37${^{\circ }}$ and aliquoted into sterile tubes containing B-27 Plus Neuronal Culture System media (A3653401, Gibco, USA)^42^ for dilution to the appropriate plating densities. Images for comparison in this study come from samples plated at low densities (10,000 and 20,000 cells/cm${^{2}}$). Following initial seeding, cells were cultured for 24 hours at 37${^{\circ }}$ after which the media was removed and replaced. Subsequently, 20% media changes were performed every three days. Samples were imaged over 20 DIV using phase-contrast microscopy (Echo Revolve 4, inverted, Discover Echo, Inc, USA) at 20X and 40X magnification.

#### Neurite image analysis

**Figure 4:**
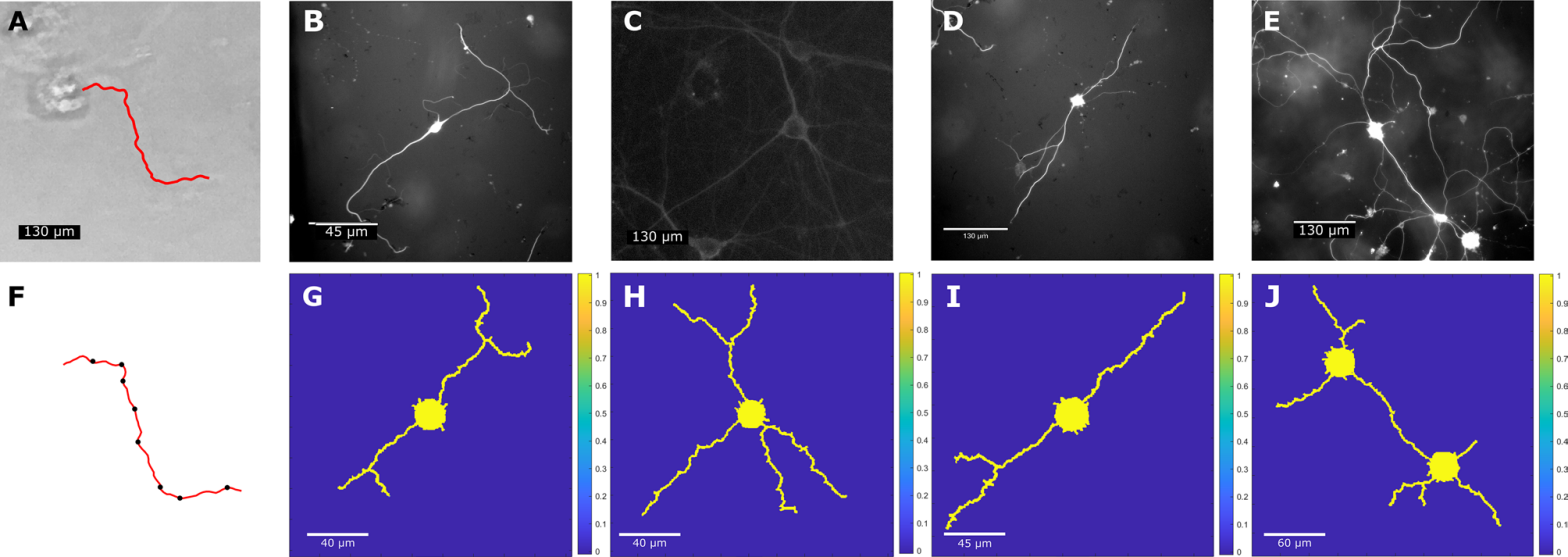
Comparison of the phase field model results of neuron growth with experimental results based on extracellular attractive cue-guided growth. (**A**) Traced neurite (red line) from a neuron cell culture at 5 DIV. (**F**) Change points (black dots) found along the neurite tracing (red line) in (**B**) using CPT algorithm with $\alpha$ of 0.05 and *q* of 2. (**B**–**E**) Experimental images of primary rat hippocampal neurons observed at 20 DIV. (**G**–**J**) Corresponding simulation results of phase field model using attractive cue-guided neuron growth on grid sizes of 443 Œ 443, 513 Œ 513, 483 × 483, and 483 Œ 483. One extracellular cue is placed at the boundary for each neurite. Bifurcation is achieved by adding more than one extracellular cue at the corresponding growth stage. The simulation results show a similar reproduction of the growth pattern. By measuring the soma diameter in experimental images with respect to their scale bars, we can evaluate scale bar units for neuron growth simulation results.

For the comparison between in vitro neurons and the growth model, neurites from the 20 DIV culture images (Fig. 4B–E,G–J were traced and segmented using the change point test algorithm (CPT)^43,44^. The turning angles of the neurites was measured and compared with the geometry of the neurites from the neuron growth model. Neurites were selected for tracing and subsequent analysis if they did not overlap with neighboring projections, were sufficiently long (greater than one soma diameter), and protruded from broad, flat somas with distinct boundaries. Once identified, the neurites were manually traced (Fig. 4A). The (X,Y) coordinates from each tracing were used to identify turns using CPT, which determined locations of significant direction changes by assessing the collinearity of vectors between consecutive pixel coordinates along the tracing. For each neurite, the CPT used a significance level ($\alpha$) of 0.05 and was run 10 times in R with the number of vectors, *q*, prior to a change point varying from 1 to 10. The lowest *q* value that resulted in the most change points was ultimately selected for analyzing the particular neurite. The neurite tracing was subsequently segmented based on the change points identified (Fig. 4F), and the length of the segments and the angle of each segment relative to the previous segment were calculated. In Fig. 4, neurites from the phase field model were also traced and then analyzed using the same CPT, and their segment lengths and turning change angles were compared with the experimental results.

The segment lengths of individual neurites and the turning angle of each segment relative to the previous segment were measured for each neurite (as shown in Fig. 4F). Table 1 shows the absolute turning angle distribution obtained for all the neurites in Fig. 4B–E,G–J. The absolute turning angle value is used for comparison with experiments because it is consistent across different time points in experiments^45^, and we can validate the effectiveness of the extracellular cue position placement to simulate neurite growth in the direction of the selected turning angle. For the 20 DIV culture images, the turning angle has a median of 32.250° with a 1st quartile (25%) of 17.206° and 3rd quartile (75%) of 56.905°. For the phase field model results, the turning angle has a median of 41.317° with a 1st quartile of 25.167° and 3rd quartile of 66.570°. Mann-Whitney analysis shows that the estimation of the median difference between culture images and phase field model results is 7.1654 with a 95.15% confidence interval for the difference between (− 9.8414, 23.4734) and a *p*-value of 0.336. This indicates that the turning angles are not statistically different between the 20 DIV neurons and the neuron growth model. Therefore CPT statistics show that the neuron growth model can reproduce similar neurite turning angle. The median segment length between each change point is 54.979 μm with an interquartile range of 70.029 μm for neuron culture image and 52.050 μm with a standard deviation of 53.425 μm for neuron growth model results.

We show the comparison between the computational model using an extracellular cue-based energy activation zone method to guide neurite extensions and experimental results from the rat hippocampal neuron culture after 20 DIV. In Fig. 4G–J, we place cues around neurons to guide neurite growth based on experimental images of neuron culture. This method can also be used to simulate multi-neuron cases with neurite interaction, as shown in Fig. 4J.

**Table 1:** Absolute turning angle of comparison cases in Fig. 4.

Parameter	${\overline{\theta }}$	$\sigma$	Minimum	1st quartile (25%)	Median	3rd quartile (75%)	Maximum
Experiment ($^{\circ }$)	41.673	32.007	6.431	17.206	32.250	56.905	154.555
Simulation ($^{\circ }$)	46.395	27.780	3.152	25.167	41.317	66.570	107.502

## Discussion

In this study, we demonstrate multiple stages of neuron growth considering the intracellular transport of tubulin. Following are our observations based on the results of the model:

- We present a new formulation of the phase field method that considers intracellular factors to model primarily stages 1 to 4 of neuron growth. In stages 1 and 2, lamellipodia formation and initial neurite outgrowth are captured in the initial few iterations followed by uniform growth of neurites. Axon differentiation (stage 3) is captured where one of the neurites differentiates into an axon with much higher growth rate as compared to the rest of the neurites. Neurites and the axon continue to grow and branch, with neurites maturing into dendrites and interacting with neighboring neurites in dendrite formation (stage 4).
- We solve the partial differential equations using the IGA collocation method. This increases the computational efficiency of the simulation while preserving accuracy.
- By adjusting the rates of assembly and disassembly of tubulin, the elongation rate of each neurite can be modeled. Thus by setting the parameters, we can automatically capture the selective growth of certain neurites leading to axon differentiation. Consumption of tubulin is enhanced further by the longest neurite due to the higher rate of assembly of free tubulin at the growing tip of the neurite. This leads to a higher elongation rate of the axon, subsequently leading to its differentiation and preventing the growth of other neurites.
- We can capture similar growth angle change of the neurites by comparing with experimental images obtained at different stages of growth by adjusting parameters based on experimental results, and easily extend the phase field method for modeling neuron networks. The growth of neurites towards each other and neurite interaction are observed. However, the model still needs to include the effect of neighboring neurons to predict accurate movement of neurites during neurite interaction and synapse formation.

There are some interesting future directions that can be included in the presented phase field model. The current model can capture different stages of neuron growth, including lamellipodia formation, initial neurite outgrowth, axon differentiation, and dendrite formation. However, in the current model, we still cannot capture the maturation stage (stage 5), where growth cone bifurcation dynamics and the formation of complex dendritic branching patterns are observed based on competitive growth. These patterns need to consider specific neuron types and their characteristic branching patterns. The model also lacks the ability to automatically capture growth stage transitions, requiring the specification of number of iterations for each stage based on experimental observations. This transition between the growth stages depends on both intra- and extra-cellular factors, in conjunction with neuron characteristics. As we work towards a more generalized mathematical model, we will focus on modeling this stage transition in the future for a particular type of neuron with a characteristic branching pattern. In the case of networks of neurons, axons move in the direction of the chemical cue, which takes place via the diffusion of chemoattractant molecules in the domain^4^. The inclusion of chemoattractant molecule-based cues into the phase field model can help in improving the accuracy of the angular variation of each neurite as compared to the current model. To improve model efficiency, we aim to carry out local refinement using truncated hierarchical B-splines to reduce computational cost while preserving geometry smoothness^46^. We plan to simulate material transport^47^ and study traffic jam^48,49^ during neuron growth in order to better model and understand neurodegenerative diseases. We also plan to investigate efficient prediction of neuron growth using data-driven approaches^50,51^.

## Methods

### Mathematical model and numerical method

In the proposed phase field model, we model the effect of intracellular factors such as tubulin to simulate neuronal development. The diffusion and active transport of tubulin from the cell body to the neurite tips as a driving force for neurite elongation is considered. We demonstrate the different stages of neuron growth by controlling the parameter values to achieve lamellipodia formation (stage 1), initial neurite outgrowth (stage 2), axon differentiation (stage 3), and dendrite formation (stage 4). For the maturation stage (stage 5), the proposed model cannot capture the biophysics of the complex dendritic tree formation that is observed in matured neurons based on different neuronal types^11,13,14^. However, the model can produce final neural geometries comparable to mature experimental neurons when driven by extracellular cues. Hence, in this paper we focus primarily on modeling stages 1–4.

#### Lamellipodia formation and initial neurite outgrowth (stages 1–2)

We formulate the phase field model based on an axonal extension model^23,41^. The phase field variable ($\phi$) is defined in the two-dimensional domain, where the value of $\phi$ is equal to 1 inside the cell and 0 in the extracellular medium. The intracellular driving factor is the tubulin concentration ($c_{tub}$) that controls neurite elongation. The phase field equation to model the initiation (stage 1) and elongation (stages 2) stages of neuron growth based on the phase field model^23,41,52^ is given as

1 $$\begin{aligned} \frac{\partial \phi }{\partial t} = M_{\phi } \left [\triangledown .(a(\Psi )^2\triangledown \phi ) - \frac{\partial }{\partial {x}} \left(a(\Psi ) \frac{\partial a(\Psi )}{\partial \Psi } \frac{\partial \phi }{\partial y}\right) + \frac{\partial }{\partial {y}} \left(a(\Psi ) \frac{\partial a(\Psi )}{\partial \Psi } \frac{\partial \phi }{\partial x}\right) + \phi (1-\phi )(\phi - 0.5 + E + 6H |\triangledown \theta |)\right], \end{aligned}$$

where $a(\Psi )$ is the gradient coefficient that models anisotropy^53^. $M_{\phi }$ is the the mobility coefficient for the phase field variable. *E* is the driving force term for phase field growth. *H* is a constant value^52^. $\theta$ indicates the change in direction of the extending neurites. We set the orientation field $\theta$ as a random value between [0, 1] in the domain, which remains fixed during the evolution of $\phi$.

**Figure 5:**
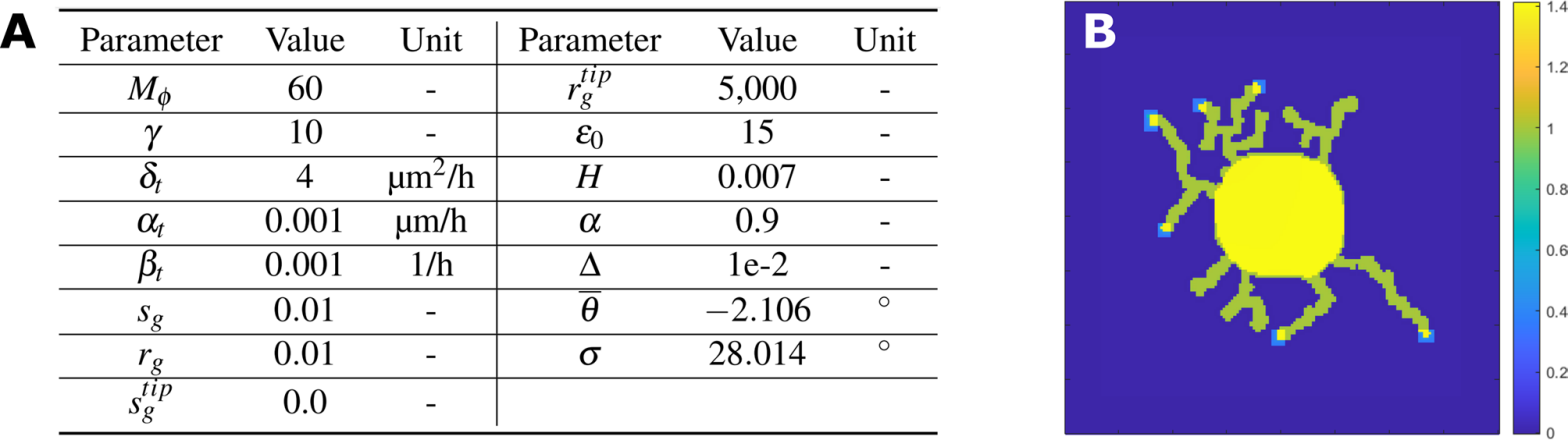
Parameter settings and simulation result showing how the phase field model is capturing neuron growth stages 1–4 using growth-cone-like activation of *E*. (**A**) Parameter settings for the phase field model. (**B**) $\phi$ overlaid with energy variable to highlighted energy activation zones.

We introduce intracellular concentration field $c_{tub}$ to evaluate neurite elongation based on tubulin concentration. Tubulin is produced inside the cell body and transported to the neurite tips by active transport and diffusion. A continuum model to simulate tubulin concentration within the growing neuron has been proposed^10,54^. A one-dimensional model is considered where the tubulin concentration can be evaluated as a continuous variable along the length of the neurite, unlike the competition model^11^, where the concentration of tubulin is only evaluated at the neurite tip. The continuum model^10^ cannot be directly extended to a 2D domain using phase field, since the phase field variable $\phi$ is defined in both intracellular and extracellular space. To ensure that $c_{tub}$ is only valid inside the growing cell, we couple the equations in the continuum model^10^ with $\phi$^55^. Thus for a moving boundary problem in 2D, we propose a new formulation to evaluate $c_{tub}$ as

2 $$\begin{aligned} \frac{\partial (\phi \,c_{tub})}{\partial t} = \delta _t \triangledown \cdot ( \phi \, \triangledown c_{tub}) - \mathbf {\alpha }_{t} \cdot \triangledown (\phi \, c_{tub}) - \beta _{t} (\phi \, c_{tub}) + \varepsilon _0 \frac{|\triangledown (\phi _0)|^2}{\int {|\triangledown (\phi _0)|^2} \, d \Omega }, \end{aligned}$$

here $\alpha _t$, $\delta _t$ and $\beta _t$ are the active transport, diffusion and decay coefficients, respectively. We introduce a new source term to include the constant production of tubulin in the cell body as $\varepsilon _0 \frac{|\triangledown (\phi _0)|^2}{\int {|\triangledown (\phi _0)|^2} \, d \Omega }$, where $\phi _0$ is the phase field variable corresponding to the initial circular cell. $\varepsilon _0$ is the dimensionless production coefficient term. We modify the definition of *E* in Eq. (1) from the axonal extension model^23^ to include the effect of change rate due to tubulin concentration. This is given as $E = \frac{\alpha }{\pi } \tan ^{-1}(H_\varepsilon (\frac{d L}{d t}) \gamma \Delta T)$, which is the driving force for cell growth. $\frac{dL}{dt} = r_{g} \, c_{tub} - s_{g}$ is the extension rate of neurites due to tubulin assembly where $r_g$ and $s_g$ are the rate constants of assembly and disassembly of tubulin^11^. $\gamma$ is the interfacial energy constant and $\Delta T$ is the undercooling temperature evolved in time^41^. Figure 5A shows the values of parameters used for the simulations. The parameter values are set empirically to capture realistic neuron geometry but can be adjusted to reflect realistic biological conditions.

In order to capture neurite morphology, we carry out “growth-cone” like activation of the driving force term *E* at the neurite tips. As shown in Fig. 5B, we consider $\phi$ field, where neurites are automatically detected using connected component analysis in MATLAB. Neurites are labeled based on the neuron to which they belong. The neurite tips are detected as points corresponding to the centroids of regional maximal $\phi$ value and having fewest neighboring points with non-zero values of $\phi$. *E* is evaluated in an energy activation zone of size $6 \times 6$ points centered at each neurite tip to allow neurite extension while setting as 0 elsewhere. We carry out self-intersection checks, where neurites corresponding to the same neuron are not allowed to intersect whereas neurite interaction between different neurons is allowed. While approaching each other, neurites having the same label are not allowed to intersect. Likewise, neurites from different neurons having different labels are allowed to intersect. As the initial condition, the cell is initialized as a circle, where we consider $\phi _0 = 1$ in the cell and $\phi _0 = 0$ in the medium (see Fig. 2A). Knot spacing in the parametric domain is set as 1, and the radius of the cell is set as $r_{0} = 20$. The simulation time step $\Delta$ is set as 0.01 . The initial normalized tubulin concentration in the cell is set using the equation $c_{tub} = \frac{1}{2}(1+ tanh((r_0-r)/2))$.

Note that our neural growth model is grid-dependent. In the $6H |\triangledown \theta |$ term of Eq. (1), the $\theta$ field is initialized differently as the resolution increases and thus introduces different values into the model, resulting in different neurite growth patterns. Because many conventional parameters used for the phase field model do not have direct physical meaning in the context of neuron growth, we followed the parameters used in the non-constraint model^23^ to develop our model. For the phase field model, because the phase transition happens in the interface region, the solution is dependent on the thickness of the interface $\delta$, which is nondimensionalized and directly defined based on the knot spacing $\Delta x$, of the parametric domain. As a result, a change in grid size will inherently change the neurite behavior in our model.

#### Axon differentiation (stage 3)

In the axon differentiation stage, competitive effects lead to a higher elongation rate of the longest neurite as compared to the other neurites^11^. The polymerization of tubulin at the ends of the neurites leads to neurite elongation. The competitive effect comes into play due to the higher consumption of tubulin by the longest neurite leading to its higher elongation rate. In the axonal extension model^23^, the modeling of the axon differentiation stage is carried out manually by setting physical constraints on the numerical model allowing only a small number of neurites to elongate and grow. This model does not consider the intrinsic factors such as tubulin concentration, thus cannot automatically constrain the growth of certain neurites. The driving force energy *E* is set as 0 for all the remaining neurites except for the axon, the longest neurite.

In order to model the axon differentiation stage, we need to incorporate the competitive effect between neurites during the tubulin assembly and disassembly process. We modify the formulation of *E* to depend on an extracellular cue-based tip selection and the length change rate of each neurite based on the tubulin concentration. The growth of certain neurites is automatically constrained to allow the extension of the axon. In the initial neurite outgrowth stage, we set the constant values of the parameters $r_g$ and $s_g$ in the entire domain such that all the neurites grow to similar lengths. For the axon differentiation stage, we need to consider different values for each neurite of $r_g$ and $s_g$ parameters to include the competitive effects^11^. The parameters $r_g$ and $s_g$ are set for each neurite at the beginning of the differentiation stage, and the selective growth of the axon is determined automatically through *E*. To achieve the axon differentiation, the longest neurite is identified and allowed to extend further by changing the values of $r_g$ and $s_g$ at the neurite tip. In order to identify the longest neurite, geodesic distance is measured from the cell center to the tip along the neurite where $\phi > 0$. Turning angles are measured for each segment of individual neurites from experimental images using the Change-Point Test (CPT)^43,44^ algorithm. A random turning angle value is selected from the normal distribution obtained using the mean turning angle ${\overline{\theta }}$ and the standard deviation $\sigma$^56^. The extracellular cue position is set at a fixed distance from the neurite tip in the direction of the selected turning angle. The energy activation zone is placed closest to the cue and $r_g^{tip}$ value is set such that $r_g^{tip} c_{tub} > s_g$, to reflect extension of the neurite thus resulting in $E>0$. We obtain $E = 0$ when $r_g^{tip} c_{tub} < s_g$, inhibiting the growth of the neurite. Thus, the axon extension process is made automatic by incorporating the intrinsic factor of tubulin concentration. As shown in Fig. 2B,C, in the initial few iterations, we can capture the stages of lamellipodia formation (stage 1) and initial neurite outgrowth (stage 2). We capture similar lengths of neurites in the initial number of iterations till the initiation stage is complete. By specifying different parameter values of $r_g$ and $s_g$ in different regions, we show the axon differentiation (stage 3) of neuron growth by including the competitive effects of neurite elongation based on tubulin concentration; see Fig. 2D.

#### Dendrite formation (stages 4)

The formation of spontaneous dendrite formation (stage 4) can also be modeled by Eqs. (1)–(2); see Fig. 2E. To capture this stage, we apply the growth-cone activation regions at the neurite tips, allowing for multiple branching geometry in neurites. Due to the flexibility of the phase field model, this method can be extended towards the simulation of neurite networks and studying the simultaneous growth of multiple neuron cells. The proposed model could be extended to capture the maturation stage of the neuron (stage 5), but it requires additional parameter tuning based on the biophysics of specific neuron type.

### Isogeometric collocation method

We utilize the isogeometric collocation method to solve the phase field equations. We solve Eqs. (1)–(2) using the multi-resolution grid approach to increase the efficiency of the collocation method. In the multi-resolution method, the domain is automatically extended by width of 10 grid points in each direction when a neurite is detected to be near the boundary of the domain. Isogeometric collocation methods directly solve the strong form of partial differential equations unlike the standard finite element approaches. They have demonstrated an overall improvement in terms of computational efficiency while still demonstrating higher order convergence^57–60^. We consider a univariate B-spline of degree *p* defined on the open knot vector $U = \{ u_1, u_2, \ldots u_{n+p+1} \}$, where *n* is the number of basis functions. For a two-dimensional domain, the bivariate basis function is the tensor product of two univariate B-splines. For all the numerical examples, we set $p=3$. We choose Greville Abscissae^61^ as the collocation points. Each collocation point ${\hat{\phi }} = \{ \hat{\phi }_u, {\hat{\phi }}_v \}$ can be written as

3 $$\begin{aligned} {\hat{\phi }}_u = \frac{\sum _{i+1}^{i+p}u}{p}, \; \mbox{and}\; {\hat{\phi }}_v = \frac{\sum _{j+1}^{j+p}v}{p}, \end{aligned}$$

where ${\hat{\phi }}_u$ and ${\hat{\phi }}_v$ are the components along each parametric direction of the collocation point ${{\hat{\phi }}}$. Equation (1) can be solved using isogeometric collocation as follows:

4 $$\begin{aligned} \frac{\partial {\hat{\phi }}}{\partial t} = M_{{\hat{\phi }}} \left[\triangledown .(a(\Psi )^2\triangledown {\hat{\phi }}) - \frac{\partial }{\partial {x}} \left(a(\Psi ) \frac{\partial a(\Psi )}{\partial \Psi } \frac{\partial {\hat{\phi }}}{\partial y}\right) + \frac{\partial }{\partial {y}} \left(a(\Psi ) \frac{\partial a(\Psi )}{\partial \Psi } \frac{\partial {\hat{\phi }}}{\partial x}\right) + {\hat{\phi }}(1-{\hat{\phi }})({\hat{\phi }} - 0.5 + E + 6H |\triangledown \theta |)\right]. \end{aligned}$$

Following the same approach, we can obtain collocated equation of Eq. (2). Directly solving the strong form of the partial differential equations reduces computational cost while maintaining the same order of accuracy and smoothness. We utilize implicit Euler time integration scheme to allow for higher time step value. We use Newton-Raphson method with a tolerance value of $1e-4$ to solve the nonlinear equations.

## Data Availability

The code and datasets generated and analysed during the current study are available in the “NeuronGrowth_IGAcollocation” repository, https://github.com/CMU-CBML/NeuronGrowth_IGAcollocation (10.5281/zenodo.5818509). Correspondence and requests for code and data should be addressed to K.Q. or Y.J.Z.
